# 

**Published:** 1869-06-01

**Authors:** 


VOL. XXVI. NUMBER 11.

### THE CHICAGO Medical Journal.

PUBLISHED SEMI-MONTHLY.

J. ADAMS ALLEN, M. D., LL. D.,
WALTER HAY, A. M., M. D.,
*EDITORS.*

**JUNE 1st.**

1869.

J. WOLCOTT HOOKER, 12 Union Building,

CHICAGO:

